# Bone Development in Children Living on Houseboats on a River in Vietnam

**DOI:** 10.2188/jea.JE2007428

**Published:** 2008-12-17

**Authors:** Tomoko Inose, Takehito Takano, Quang Khac Luong Nguyen, Keiko Nakamura, Masafumi Watanabe, Kaoruko Seino

**Affiliations:** 1 Health Promotion, Graduate School of Tokyo Medical and Dental University, Tokyo, Japan; 2 International Health, Graduate School of Tokyo Medical and Dental University, Tokyo, Japan; 3 Hue Healthcare Center, Hue, Vietnam

**Keywords:** Bone Development, Child, Living on Boats, Quantitative Ultrasonometry, Socioeconomic Factors

## Abstract

**Background:**

With the rapid urbanization of Vietnam, living on boats has come to be associated with underprivileged socioeconomic status, and there are major concerns regarding the health of children living under such conditions. Bone development is a critical concern in children because the foundation for skeletal health is established early in life. We evaluated the bone properties of children living under underprivileged conditions on boats in Hue City, Vietnam, with respect to a number of household factors.

**Methods:**

One hundred and twenty children aged 7-11 years selected randomly from households living on boats in Hue were included in this study. Tibial and radial speed of sound (SOS) were measured by non-invasive quantitative ultrasonometry. Socioeconomic profiles, health promotion proficiency, and nutritional intake of households were assessed by structured interview. The bone properties of 60 children aged 7-11 living on land were also assessed as controls.

**Results:**

The Z-scores of tibial and radial SOS of boat children were -0.16 ± 0.89 and -0.24 ± 0.75 (mean ± SD), respectively. Tibial SOS exhibited a significant correlation with radial SOS (r = 0.39, *P* < 0.01). Among the household factors examined, the educational level of fathers (*P* < 0.05) and the health promotion proficiency (*P* < 0.05) of households exhibited positive associations with the tibial bone properties of the children. The tibial and radial SOS of boat children were lower than those of children living on land (*P* = 0.001 and *P* = 0.086, respectively).

**Conclusion:**

The results of the present study revealed the underdevelopment of bone properties in children living on houseboats, which was correlated with their living conditions.

## INTRODUCTION

Living on boats along rivers is a common traditional way of life in Vietnam. Currently, there are 941 households dwelling on houseboats along the Huong River in Hue City, Vietnam, which provides a convenient location for both work and life on the water.^1^ People living on the river are employed mainly in sand extraction, fisheries, transportation, or other work on land.^1^^,^^2^ People living on boats live, cook, and sleep on houseboats, some of which are moored along the banks of the river, while others are moving on the river.^1^

However, with urbanization, living on boats has come to be associated with underprivileged living conditions;^3^ people living on boats are underprivileged with regard to health, socioeconomic status, sanitary conditions, access to clean drinking water, household spaces, and other living conditions.^1^^,^^2^ The majority of such households on the river include two or three generations living together with many children, and therefore cramped living conditions are prevalent.^1^ A great deal of attention has focused on how living under such conditions on boats can influence the growth and development of children.

Bone development status is a critical indicator of lifelong bone health, because the foundation of skeletal health is established early in life.^4^^,^^5^ The magnitude of peak bone mass is influenced by lifestyle-related factors, such as physical activity and nutritional intake in preadolescence, as well as by specific genetic factors.^4^^,^^5^ Healthy bone development is a critical concern in children living under cramped, underprivileged conditions on houseboats, as preadolescence is a crucial period for lifelong bone health.

The present study was performed using quantitative ultrasonometry (QUS), a radiation-free method that measures the speed of sound (SOS) along cortical bones. This is a well-established method that is increasingly being applied for the assessment of bone properties in children.^6^^-^^9^ SOS reflects both qualitative and quantitative properties of bone, including cortical density and thickness.^10^^-^^12^

In the present study, we evaluated the development of bone properties using the QUS method in children living under low socioeconomic conditions in underprivileged living environments on houseboats on the Huong River, Hue City, Vietnam. The objectives of the study were to determine the developmental status of bone properties of children living on boats and to evaluate the associations between bone properties and household factors.

## METHODS

### Subjects

The study population comprised children aged 7-11 years living on houseboats on the Huong River and an age-matched control group consisting of children living on land in Hue City, Vietnam. The families of children on a randomly selected list of children aged 7-11 living on boats obtained from the community household registration records were asked to participate in the study in consecutive order of their appearance on the list. The requests were repeated until 120 children completed the examination (a total of 150 children, 18% of all children aged 7-11 living on boats in Hue, were required to acquire measurements of 120 children). The families of children on a randomly selected list of those aged 7-11 living on land obtained from the student records of a primary school located close to the Huong River were also asked to participate in consecutive order of their appearance on the list. The requests were repeated until 60 children completed the examination (a total of 62 children, 15% of all children from this school and 0.9% of all primary school children living on land along the Huong River were required to acquire the measurements of 60 children). According to a report by the municipal government, the school attendance rate of all children in Hue City aged 7-11 was 98.6%.^13^ Therefore, we used the student records of a school as a reliable list of children living on land. There were no children from the same households among the subjects included in this study. We recruited twice as many children from boat households as those living on land. The necessary sample size was calculated to have an 80% chance of detecting a significant difference (α = 0.05, two-sided), on the assumption of a difference in mean Z-score of 0.5 and a variance of 0.7. According to the above calculation, a minimum of 48 children of each sex living on boats and 24 children of each sex living on land were required. We enrolled 120 children living on boats and 60 children living on land. Permission to implement this study with consideration of its ethical appropriateness was obtained from the Hue People’s Committee. Informed consent for participation in this study was obtained from all children and their guardians.

### Quantitative Ultrasonometry

Bone properties expressed as SOS were measured at the mid-shaft of the tibia and the distal third of the radius by quantitative ultrasonometry (Sunlight Omnisense 7000P). A single well-trained operator performed measurements in all the subjects. Intra-operator variation had been tested previously, and the coefficient of variation was found to be less than 0.4%. Instrument-derived Z-scores based on the age- and sex-adjusted values of tibial and radial SOS were determined using Asian pediatric reference curves (Sunlight Omnisense 7000P). The Z-score is the difference between the subject’s SOS result and the average SOS of a population of the same age and sex in units of population standard deviation. For example, a Z value of +0.5 indicates that the subject’s SOS is half the population standard deviation above the mean of his or her age-matched peers.

### Questionnaire Survey

A household questionnaire survey was conducted on the boats where the children lived. Information was obtained by interview teams in face-to-face interviews with the head of the household (i.e., the child’s father or mother). Each team consisted of one physician, one nurse, and one civil servant. The questionnaire was first developed in English and then translated into Vietnamese. The consistency of expressions in English and Vietnamese was examined by back-translation from Vietnamese to English. In order to conduct standardized interviews, the interview team members participated in a training workshop prior to conducting the survey. The validity and reliability of the questionnaire were tested in practice visits to boat households by the interview teams. The following items were assessed.

*Socioeconomic profile*: Household monthly income and occupation, the number of family members, the child’s school attendance, highest attainment in formal education and literacy of fathers and mothers, and the number of boats per household. Monthly income per capita was calculated by dividing household monthly income by the number of household members. The children were divided into quartiles based on the income per capita.

*Health promotion proficiency*: We developed a “health promotion proficiency score” of the households based on participation in health education programs, health practices in the home, and health knowledge, which were assessed based on the questions listed in the Appendix. The questions were developed by reference to the reported health issues among boat households^1^ and a key informant interview with 6 physicians and 6 community leaders. The health promotion proficiency score was categorized as low (score, 0-1), medium (score, 2-4), or high (score, 5-9).

*Nutritional intake*: The number of days per week on which the families ate meat, fish, vegetables, eggs, dairy products, rice, noodles, and bread.

### Height and Weight

Height (cm) and weight (kg) were measured using a wall-mounted stadiometer and a non-electric weight meter, respectively. Body mass index (BMI) was calculated as weight/height squared (kg/m^2^). Height for age, weight for age, and BMI for age were calculated according to the National Center for Health Statistics, Centers for Disease Control and Prevention growth chart.^14^

### Statistical Analyses

Means ± standard deviations (SDs) of tibial and radial SOS of the children living on boats were calculated by both age and sex. Differences in the Z-scores of tibial and radial SOS between boys and girls were tested using independent samples t-tests. The associations between tibial and radial SOS were assessed based on Pearson’s correlation coefficient. In order to assess the association between household factors and bone properties of the children in different age groups, the Z-score of SOS was used for the analyses. The means ± SDs of the Z-score of SOS were calculated according to socioeconomic profiles and health promotion proficiency score, and compared using a one-way analysis of variance (ANOVA) for income per capita, occupation of households, and health promotion proficiency score, and differences were tested using independent samples t-tests for children’s school attendance, highest attainment in formal education and literacy of fathers and mothers, and the number of boats owned. Multivariable linear regression analyses were performed to evaluate the independent effect of the father’s highest formal educational attainment on bone properties after adjustment for the occupation of households, and income per capita. Multivariable linear regression analyses were performed to evaluate the independent effect of health promotion proficiency on bone properties after adjustment for the father’s highest educational attainment, occupation of the household, and income per capita. The father’s highest educational attainment was used for multivariable linear regression analyses, as this was highly correlated with the mother’s highest educational attainment and the literacy of fathers or mothers. Differences in the mean monthly income per capita by categories of the number of boats of the households were compared using independent samples t-tests. The mean numbers of days per week on which the families ate each type of food were also calculated. The associations between the number of days on which they ate each type of food and the Z-score of SOS were assessed based on Spearman’s correlation coefficient. Differences in the Z-score of tibial and radial SOS, and the height, weight, and BMI for age between the children living on boats and those living on land were tested for significance using independent samples t-tests. Statistical analyses were performed using SPSS^®^ 15.0J for Windows.

## RESULTS

Table 1 shows the means and SDs of the tibial and radial SOS of children living on boats according to age and sex. Shallow increases in SOS with age at both the tibia and radius were observed. The mean Z-score of tibial SOS of boys was significantly higher than that of girls (*P* < 0.05). The tibial SOS of children living on boats exhibited a significant correlation with radial SOS (r = 0.39, *P* < 0.01).

**Table 1.** Tibial and radial speed of sound (SOS) of children living on boats (n = 120)

	Age (years)	Tibia				Radius	
	
		Boys		Girls		Boys	Girls
			
		n	Mean ± SD	n	Mean ± SD	Mean ± SD	Mean ± SD
SOS (m/s)	7	11	3487 ± 138	8	3506 ± 47	3597 ± 65	3639 ± 83
	8	8	3517 ± 93	8	3453 ± 120	3649 ± 78	3643 ± 99
	9	15	3524 ± 94	4	3587 ± 17	3677 ± 74	3653 ± 116
	10	17	3556 ± 73	24	3504 ± 104	3682 ± 60	3658 ± 91
	11	13	3571 ± 75	12	3527 ± 85	3699 ± 89	3718 ± 53

Z-score of SOS	All	64	0.03 ± 0.90	56	-0.38 ± 0.84 *	-0.25 ± 0.69	-0.23 ± 0.82
		-0.16 ± 0.89				-0.24 ± 0.75	

SD: standard deviation

^*^P < 0.05: Difference between boys and girls (independent samples t-tests)

Table 2 shows the Z-scores of the tibial and radial SOS of children living on boats according to socioeconomic profiles and health promotion proficiency. There were no significant correlations between whether the children went to school or not and their bone properties. The educational level and literacy of fathers were both significantly associated with the children’s tibial SOS; the same trend was also observed with regard to the tibial and radial SOS in boys and girls. The educational level of fathers contributed significantly to the Z-score of tibial SOS after adjustment for the occupation of households and income per capita. The radial SOS was lower in children of households without an occupation than in those children whose household occupation was fishing; however, the associations between household occupation and bone properties were different for the tibia and radius and between boys and girls. The Z-scores of tibial and radial SOS of children whose households had one boat tended to be lower than those of the children whose households had two or more boats, and the difference was statistically significant in the radial SOS in boys. There were no statistically significant differences between the mean monthly income per capita of households with one boat and those with two or more boats. A higher health promotion proficiency score was significantly related to a higher Z-score of the children’s tibial SOS. Health promotion proficiency contributed significantly to the Z-score of tibial SOS after adjustment for the highest formal educational attainment of fathers, occupation of households, and income per capita.

**Table 2.** Socioeconomic profiles and health promotion proficiency scores of households and Z-scores of tibial and radial speed of sound (SOS) of children living on boats

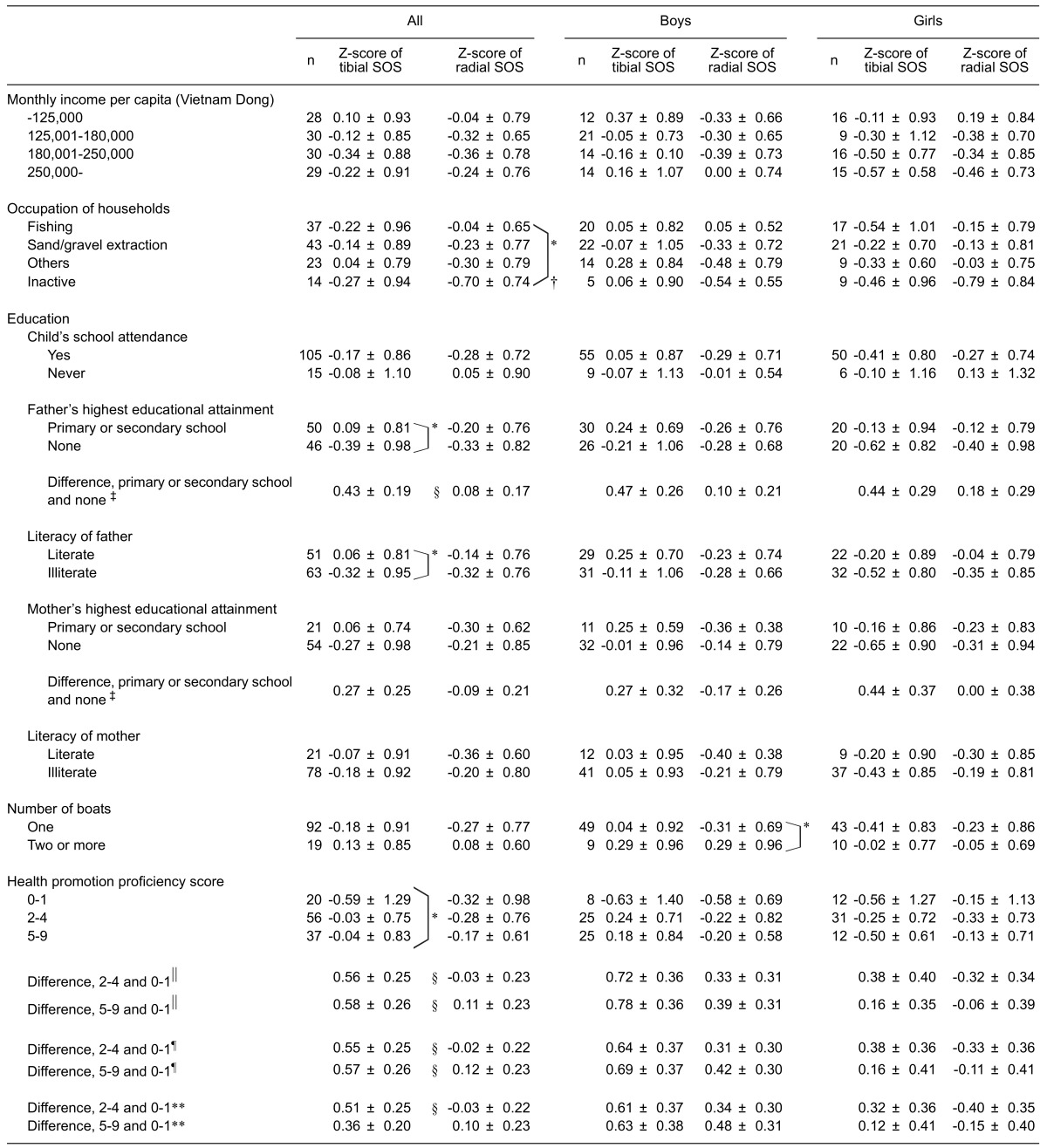

Values are means ± standard deviation.

*: *P* < 0.05: comparing the Z-score of the SOS among groups using a one-way analysis of variance (ANOVA) for income per capita, occupation of household, and health promotion proficiency score, and using independent samples t-tests for education and number of boats.

†: *P* < 0.05. difference between Z-scores of SOS of fishing and inactive groups by Bonferroni’s t-test

‡: Differences between the Z-scores of the SOS of children whose father’s or mother’s highest educational attainment was primary or secondary school and those of children whose father or mother had never attended school using multivariable linear regression analysis adjusting for income per capita and occupation of households.

§: *P* < 0.05

||: Differences between the Z-scores of the SOS of children whose health promotion proficiency was 2-4 or 5-9 and those of children whose health promotion proficiency was 0-1 using multivariable linear regression analysis adjusting for the highest attainment in formal education of fathers.

¶: Differences between the Z-score of the SOS of children whose health promotion proficiency was 2-4 or 5-9 and those of children whose health promotion proficiency was 0-1 using multivariable linear regression analysis adjusting for the highest attainment in formal education of fathers and occupation of households.

**: Differences between the Z-score of the SOS of children whose health promotion proficiency was 2-4 or 5-9 and those of children whose health promotion

proficiency was 0-1 using multivariable linear regression analysis adjusting for the highest attainment in formal education of fathers, occupation of households, and income per capita.

The mean numbers of days per week on which the families ate each type of food were as follows: 2.3 for meat, 4.8 for fish, 4.5 for vegetables, 1.2 for eggs, 0.4 for dairy products, 6.7 for rice, 1.9 for noodles, and 2.4 for bread. Table 3 shows the associations between the numbers of days per week on which the families ate each type of food and the Z-scores of SOS of the children living on boats. The number of days per week on which the families ate vegetables was correlated with the Z-score of tibial SOS of the children living on boats.

**Table 3.** Spearman correlation coefficient between number of days per week on which the families ate each type of food and the Z-scores of the tibial and radial speed of sound (SOS) of children living on boats

Number of days per week the families ate	Z-score of tibial SOS			Z-score of radial SOS		
	
	All	Boys	Girls	All	Boys	Girls
Meat	-0.01	0.01	-0.13	-0.12	0.05	-0.26 *
Fish	0.01	0.04	0.01	0.04	0.03	0.06
Vegetables	0.19 *	0.09	0.21	-0.13	-0.21	-0.07
Eggs	0.02	0.04	-0.01	0.03	0.04	0.03
Dairy products	0.02	-0.04	0.02	-0.13	-0.23	0.01
Rice	0.03	0.08	-0.01	-0.13	-0.05	-0.23
Noodles	-0.06	-0.02	-0.14	-0.08	-0.13	-0.04
Bread	-0.04	-0.13	0.08	0.05	-0.02	0.11

*: *P* < 0.05

Table 4 shows the Z-scores of tibial and radial SOS, and the height, weight, and BMI for age of the children living on boats and those living on land. The Z-scores of tibial and radial SOS of boat children were lower than those of the children living on land (*P* = 0.001 and *P* = 0.086, respectively). Children living on boats exhibited lower values for indices of bone development as compared with those living on land. The means and SDs of tibial and radial SOS of children living on land according to age and sex are shown in Table 5.

**Table 4.** Z-scores of the tibial and radial speed of sound (SOS), and the height, weigh, and body mass index (BMI) for age of children living on boats and those living on land

	Children living on boats						Children living on land		
	
	All (n = 120)		Boys (n = 64)		Girls (N = 56)		All (N = 60)	Boys (N = 27)	Girls (N = 33)
Z-score of tibial SOS	-0.16 ± 0.89	**	0.03 ± 0.90	**	-0.38 ± 0.84	*	0.30 ± 0.85	0.60 ± 0.77	0.05 ± 0.85
Z-score of radial SOS	-0.24 ± 0.75		-0.25 ± 0.69		-0.23 ± 0.82		-0.04 ± 0.77	0.04 ± 0.70	-0.10 ± 0.83
Height for age †	-2.26 ± 0.97	***	-2.31 ± 0.92	***	-2.20 ± 1.04	***	-0.94 ± 1.02	-0.86 ± 0.88	-0.99 ± 1.13
Weight for age †	-2.52 ± 1.31	***	-2.65 ± 1.41	***	-2.38 ± 1.18	***	-1.12 ± 1.24	-0.87 ± 1.45	-1.32 ± 1.01
BMI for age †	-1.42 ± 1.56	*	-1.48 ± 1.58	**	-1.35 ± 1.54		-0.85 ± 1.30	-0.42 ± 1.53	-1.22 ± 0.96

Values are means ± standard deviation.

*P < 0.05, **P < 0.01, ***P < 0.001: Differences between children living on boats and those living on land using independent samples t-tests.

†: Calculated according to National Center for Health Statistics, Centers for Disease Control and Prevention growth chart.

**Table 5.** Tibial and radial speed of sound (SOS) of children living on land (n = 60)

	Age (years)	Tibia				Radius	
	
		Boys		Girls		Boys	Girls
			
		n	Mean ± SD	n	Mean ± SD	Mean ± SD	Mean ± SD
SOS (m/s)	7	7	3589 ± 56	7	3497 ± 95	3687 ± 73	3632 ± 65
	8	4	3546 ± 109	8	3513 ± 85	3664 ± 64	3647 ± 124
	9	7	3621 ± 96	6	3587 ± 111	3714 ± 105	3699 ± 83
	10	8	3587 ± 75	12	3597 ± 86	3697 ± 65	3703 ± 74
	11	1	3701	0		3731	

Z-score of SOS	All	27	0.60 ± 0.77	33	0.05 ± 0.85 ^*^	0.04 ± 0.70	-0.10 ± 0.83
		0.30 ± 0.85				-0.04 ± 0.77	

SD: standard deviation

*P < 0.05: Difference between boys and girls (independent samples t-tests).

## DISCUSSION

The bone properties of the children living on houseboats in Hue City, Vietnam, were examined with respect household factors. The Z-scores of SOS of boat children indicated a significant underdevelopment of bone properties in these children. Among the household factors examined, the tibial bone properties of the children exhibited a significant association with the educational level of their fathers and health promotion proficiency. Bone properties were also related to other household factors, although most of these associations were not statistically significant.

Almost all the households living on boats in the area were included in the household registration records from which the subjects were drawn at random. Therefore, the subjects included in this study were regarded as a representative sample of children living on boats in Hue City. The children in this study were in preadolescence, the period during which the effects of hormones on bone properties are relatively small and both lifestyle factors and the living environment are known to be the major determinants of bone development.^4^^,^^5^ The results of the present study revealed evidence of bone underdevelopment in those children living under low socioeconomic and underprivileged conditions on boats, and indicated correlations between this skeletal underdevelopment and lifestyle-related factors. As bone mass accumulation is not uniform at all sites,^7^ in this study, the associations between children’s skeletal development and lifestyle-related factors were examined in different skeletal regions.

The bone properties of the tibia in boat children with low socioeconomic status exhibited significant associations with the educational level and literacy of their fathers; the tibial SOS of children whose fathers had attended primary or secondary schools were significantly higher than those of children whose fathers had never attended school. The association held even after adjustments for the occupation of households and income per capita. Given this observation, it is suggested that an enhancement of the educational level of the population could be an important social determinant for healthy bone development in children. With regard to the relationships between the children’s bone properties and both household income and occupation, the associations were different for the tibia and radius and between boys and girls. The lack of clear associations with regard to bone properties and the income or occupation of households could be accounted for by unstable working conditions among the boat households as well as the relatively narrow range of variation in income.^1^

In the present study, the bone properties of the children whose households had only one boat tended to be lower than those of children whose households had two or more boats. Although there was no significant association between the number of boats per household and household income, the number of boats per households may reflect better overall socioeconomic living conditions of households, including their ability to make a living. Further studies are required in order to determine the effects of the number of boats owned as well as the household lifestyle on the bone development of children.

The health promotion proficiency score adopted in the present study was an integrated indicator reflecting both knowledge and skills related to health promotion. The results of the present study indicated that the health promotion proficiency of households is positively associated with the bone development of children living under low socioeconomic and fragile conditions on boats. The effects of health promotion proficiency on bone properties of the children were significant after adjustment for the education of fathers as well as household occupation and per capita income. Programs to increase health promotion proficiency through providing information, health education, and enhancing the life skills of people living on boats will contribute to the healthy development of children living under these conditions.

Of the variety of foods assessed in the present study, only vegetable intake was positively correlated with the children’s bone properties. This was considered to be due to the low intake of food among the boat households in general, which resulted in no associations between intake of food in most categories and bone development in the children living on boats. A previous study performed in Mali^15^ suggested a positive correlation between dietary diversity and household socioeconomic status, and a study that analyzed demographic and health surveys from 11 countries indicated a positive association between dietary diversity and nutritional status.^16^ Anthropometric indices of children living under low socioeconomic conditions on boats indicated their poor nutritional status; values of less than -2 for height for age, weight for age, and BMI for age are referred to as stunting, underweight, and wasting, respectively.^17^^-^^19^ Bounds et al.^20^ reported that the longitudinal intakes of many nutrients from age 2 months to 8 years are related to bone mineral contents at 8 years of age. Longitudinal low nutritional status is likely to play a role in impeding the bone development of children living on boats.

With the rapid urbanization of Vietnam, the traditional way of life involving living on boats has come to be associated with underprivileged socioeconomic status.^1^^,^^2^ The results of this empirical study indicate reduced development of bone properties in children belonging to such boat households in Hue that are characterized by low socioeconomic status, low nutritional status, and fragile living conditions. Although a lack of information on the socioeconomic status of the subjects living on land could be considered as a limitation of this study, the investigation of household factors pertaining to boat children does indicate that the educational level of fathers and the health promotion proficiency of households were the factors that positively contribute to children’s bone development. Further studies that take into consideration the significance of the influence of socioeconomic factors and factors related to life on boats on bone properties would broaden our understanding of the reduced bone development in children living on boats.

In conclusion, the bone properties of children from boat households in Hue City, Vietnam, were examined, and the results indicated reduced skeletal development in these children. The educational level of fathers and the health promotion proficiency of boat households were positively associated with the bone properties of children living under underprivileged conditions on boats. These empirical findings will contribute to the development of effective health promotion programs with a broader and more comprehensive approach that take into consideration the entire spectrum of factors affecting children living under fragile conditions.

## Appendix. Questions to assess the health promotion proficiency of households.

**Table:**

Questions	Scores
Have you ever participated in programs that provide health and sanitation education? (Yes; No)	Yes: (1), No: (0)
Do you wash your hands before cooking and eating? (Yes; No)	Yes: (1), No: (0)
Do you make foods hot enough before eating? (Yes; No)	Yes: (1), No: (0)
Name at least four parasitic worms.	Named 0: (0), One: (1), More than two: (2)
Do you know at least two means to prevent infectious diseases?	Don’t know: (0), Know one: (1), Know two: (2)
Do you know at least two means to prevent malnutrition?	Don’t know: (0), Know one: (1), Know two: (2)

The total score of answers to all the above questions was defined as the health promotion proficiency score.
